# The prevalence of pre-hypertension and its association to established cardiovascular risk factors in south of Iran

**DOI:** 10.1186/1756-0500-5-386

**Published:** 2012-07-28

**Authors:** Karamatollah Rahmanian, Mohammad Shojaie

**Affiliations:** 1 Department of Social Medicine, Jahom University of Medical Sciences, Jahrom, Iran; 2 Department of Internal medicine, Jahom University of Medical Sciences, Motahhari Avenue, Jahrom, Iran

**Keywords:** Pre-hypertension, Cardiovascular risk factor, Obesity, BMI

## Abstract

**Background:**

Pre-hypertension is associated with an increased risk of the development of hypertension and subsequent cardiovascular disease and raises mortality risk. The aim of this study was to determine the prevalence of pre-hypertension and to explore the associations between pre-hypertension and established cardiovascular risk factors in a population-based sample of Iranian adults.

**Methods:**

In this cross-sectional study a representative sample of 892 participants aged ≥30 years was selected using a multistage cluster sampling method. After completion of a detailed demographic and medical questionnaire (gender, age, history of diabetes mellitus and hypertension, taking antihypertensive or hypoglycemic agents and history of smoking), all participants were subjected to physical examination, blood lipid profile, blood glucose, anthropometric and smoking assessments, during the years 2009 and 2010. Variables were considered significant at a p-value ≤ 0.05. Statistical analysis was performed using SPSS version 11.5 software.

**Results:**

Pre-hypertension was observed among 300 (33.7%) subjects, 36.4% for men and 31.4% for women (p > 0.05). The pre-hypertensive group had higher levels of blood glucose and triglycerides, higher body mass index and lower percentage of smoking than did the normotensive group. Multivariate logistic regression analysis showed that obesity and overweight were the strongest predictors of pre-hypertension [odds ratio, 2.74: 95% CI (Confidence Interval), 1.62 to 4.62 p < 0.001; odds ratio, 2.56, 95% CI, 1.74 to 3.77, p < 0.001 respectively].

**Conclusions:**

Overweight and obesity are major determinants of the high prevalence rate of pre-hypertension detected in Iranian population. Therefore, primary prevention strategies should concentrate on reducing overweight and obesity if the increased prevalence of pre-hypertension is to be diminished in Iranian adults.

## Background

High blood pressure is an important subject in primary care in the 21st century and has become growing worldwide, especially in developing countries [1]. Furthermore, hypertension is one of the most important health problems because of its relation to ischemic heart disease, which is the leading cause of death [2]. The prevalence of hypertension is 8-14% in population, worldwide [3]. In Iran, the Iranian Health Profile Survey (IHPS) of 1999 reported a hypertension prevalence of 12.54% in adults older than 30 years [4]. High blood pressure and its complications is a significant problem and cardiovascular diseases are the most common cause of death in Iran [5]. Many of studies on different populations have identified certain factors such as older age, hypercholesterolemia, diabetes mellitus and increased body mass as being associated with high blood pressure [2,6,7]. The last years, some researchers have documented an increase in the risk of cardiovascular disease in subjects with systolic blood pressures (SBP) between 120 – 139 mmHg and/or diastolic blood pressures (DBP) between 80 – 89 mmHg [8,9]. Based on this current evidence, the Seventh Report of the Joint National Committee (JNC7) on Prevention, Detection, Evaluation and Treatment of High Blood Pressure recommended a new classification of blood pressure level under the term pre-hypertension [6].

Pre-hypertension is associated with an increased risk of the development of hypertension and subsequent cardiovascular disease and raises mortality risk [10-13]. There are additional reports of prevalence percentages for pre-hypertension, with estimates ranging from 30 – 48.9% [6,7].

The aim of the present study was to estimate the prevalence of pre-hypertension and to examine its association with established cardiovascular risk factors (i.e. overweight, obesity, hypercholesterolemia, diabetes mellitus and smoking) from a population-based study of Iranian adults, in order to propose primary preventive strategies for combating it.

## Methods

### Study participants

The present investigation adopted a cross-sectional design protocol. The population studied - a multi-stage stratified clustering sample – was derived from a patient cohort from ten urban health centers operating in Jahrom City in the South region of Iran. Pregnant and lactating women and/or persons with chronic disease and mental disorders and unable to walk, were excluded. The final sample consisted of 405 males (aged 51.9 ± 13.9 years) and 487 females (aged 48.5 ± 12.9 years). All subjects (892) answered a demographic and a detailed medical questionnaire.

Afterwards, they were subjected to anthropometric, blood pressure and fasting blood glucose and lipid profile measurements. All data were collected during the years 2008 and 2009.

Identical standard protocols were used for each measurement conducted by trained physician. Signed informed sanction was obtained from all participants and Ethical approval was obtained from the Ethics Committee of the Jahrom University.

### Instruments and measurements

#### Demographic and medical questionnaire

This includes the demographic and medical records of patients about “gender, age, history of diabetes mellitus and hypertension, taking antihypertensive or hypoglycemic agents.

#### Smoking status assessment

Smoking status was ascertained by means of a questionnaire. Subjects, who smoked one or more cigarettes or one cup of water pipe per week, were considered as smokers.

#### Anthropometry

Body weight was measured (Seca 700, Germany) to the nearest 100 grams, with light cloth and without shoes. Height was measured to the nearest 0.5 cm, without shoes using a stadiometer (Seca 700, Germany). Body mass index (BMI) was calculated as weight (in kilograms) divided by height (in meters) squared.

#### Blood pressure

Blood pressure was measured after the subject had rested for at least 5 minutes and from right arm placed at the heart level by a physician. Two measurements were taken by a mercury sphygmomanometer (Richter, Germany) with at least 5 minutes between successive measurements. The mean of two measurements of Korotkoff phase I was recorded for systolic blood pressure (SBP). The mean of two values of Korotkoff phase IV was recorded for diastolic blood pressure (DBP). Pre-hypertension was defined as having either a SBP of 120 to 139 mmHg and/or DBP of 80 to 89 mmHg, according to JNC7, in persons who were not on antihypertensive medication [14]. Hypertension was defined as an average SBP ≥140 mmHg, an average DBP ≥ 90 mmHg, and/or self reported current treatment for hypertension with antihypertensive medication.

#### Serum assessments

Venous blood was collected in the morning after an overnight fast and serum was used for the biochemical tests. Diabetes mellitus was defined as having two fasting serum glucose assessment of equal to or more than 126 mg/dl or being on treatment for diabetes [15]. Dyslipidemia was defined according to ATP III report [16]. Hypercholesterolemia was defined as fasting total serum cholesterol of greater than or equal to 240 mg/dl. Blood concentration of LDL-C (low-density lipoprotein cholesterol) equal or above 160 mg/dl and blood concentration of HDL-C (high-density lipoprotein cholesterol) under 40 mg/dl respectively, were considered to be undesirable.

### Statistical analysis

For statistical analysis the BMI (kg/m^2^) was classified into two categories: overweight: 25–29.9 and obese: ≥30.0. Continuous variables were presented as mean values and standard deviation. Categorical variables were presented as frequencies. Associations between categorical variables were tested by the use of contingency tables and the chi-square test. Independent *T*-test was used to comparisons between continuous variables and blood groups (hypertension or pre-hypertension with normal blood pressure). Binary logistic regression was used to assess the association of age, gender, smoking and anthropometric and serum parameters to blood pressure (pre-hypertension or normal) by estimating the odds ratio with 95% confidence interval (CI). Variables were considered significant at a p-value ≤ 0.05. Statistical analysis was performed using SPSS version 11.5 software.

## Results

### Characteristics of study participants

A high percentage prevalence of pre-hypertension was observed in the studied population, 33.7% (95% confidence interval [CI]; 30.6%-36.8%). Pre-hypertension was higher in men than in women (36.4%; CI, 31.7%-41/1% and 31.4%; CI, 27.3%-35.5%) but there was no significant difference in the percentages (p > 0.05). The prevalence rate of hypertension among all subjects was 35.4%. However, no significant difference was found in the percentages of the prevalence rate of hypertension in male (35.1%) and female (35.5%) participants (p > 0.05).

**Figure 1:**
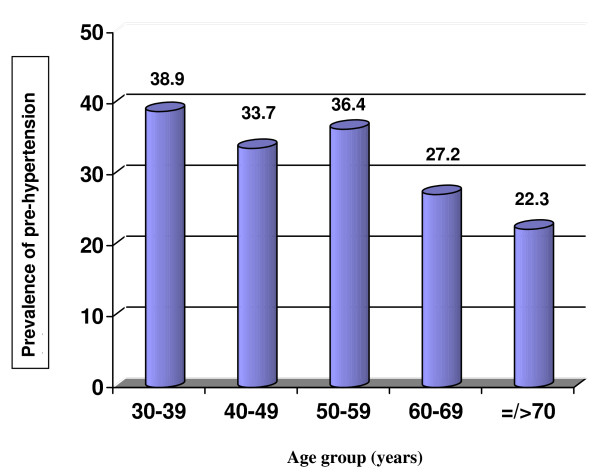
Prevalence of pre-hypertension by age groups.

Figure 1 illustrates an age-group specific pattern concerning the pre-hypertension prevalence rate. A steady decline in the prevalence of pre-hypertension is apparent with an exception for the 50–59 age group (p = 0.025).

In Table 1 are presented the epidemiological characteristics of the study participants as blood pressure groups. It appears that fasting serum glucose, triglyceride and BMI mean values were higher in pre-hypertensive group than in the normotensive group, respectively (p = 0.013; p = 0.002; p < 0.001).

**Table 1:** Subjects characteristics by blood pressure group. Figures are Mean (±SD)

**Variable**	**Normotension**	**pre-hypertension**	p*	**hypertension**	p**
Age (years)	43.8 ± 10.0	48.1 ± 12.8	<0.001	57.1 ± 13.4 < 0.001	<0.001
Glucose^#^	88.9 ± 27.1	94.8 ± 29.5	0.013	109.4 ± 50.7	<0.001
Cholesterol^#^	184.9 ± 41.2	188.7 ± 37.9	0.225	199.0 ± 44.5	<0.001
LDL cholesterol^#^	112.7 ± 28.6	113.6 ± 30.1	0.704	119.8 ± 35.6	0.008
HDL cholesterol^#^	47.4 ± 10.8	47.5 ± 10.3	0.851	48.5 ± 10.5	0.195
Triglyceride^#^	121.9 ± 73.2	144.7 ± 98.6	0.002	155.9 ± 88.8	<0.001
BMI, kg/m^2^	25.3 ± 4.3	26.8 ± 4.3	<0.001	26.9 ± 4.4	<0.001

* Pre-hypertensive to normotensive subjects comparisons.

** Pre-hypertension to normotensive subjects comparisons.

# mg/dl.

In Table 2 we can observe that the pre-hypertensive group exhibited higher prevalence of overweight (p < 0.001) and its subjects had 1.77 times increased probability of being overweight than their normotensive counterparts. Furthermore the preh-ypertensive group had higher prevalence of diabetes mellitus, total hypercholesterolemia, and obesity but there was no statistical difference in percentages (p > 0.05). In addition, fewer pre-hypertensive subjects reported smoking than their normotensive counterparts (12.3% vs 18.8%; p = 0.031).

**Table 2:** Prevalence of individual cardiovascular risk factors (%) by blood pressure group

**Variable**	**normal**	**pre-HTN**	RR* (95% CI)	**HTN**	**RR**^**#**^**(95% CI)**
Sex (male,%)	41.7	49.0	1.34 (0.97-1.87)	45.1	1.15 (0.83-1.59)
Smoking	18.8	12.3	0.6 (0.38-0.96)	8.6	0.40 (0.24-0.66)
Diabetes mellitus	4.7	8.7	1.92 (0.97-3.81)	20.6	5.26 (2.83-9.78)
Total	7.6	10.0	1.35 (0.75-2.42)	16.5	2.40 (1.40-4.10)
Hypercholesterolemia					
High LDL level	5.4	6.3	1.18 (0.58-2.36)	12.7	2.53 (1.36-4.69)
Low HDL level	9.1	9.0	0.99 (0.56-1.76)	7.3	0.79 (0.43-1.42)0
Hypertriglyceridemia	11.6	16.7	1.52 (0.95-2.46)	24.4	2.46 (1.57-3.86)
Overweight	35.1	49.0	2.15 (1.53-3.01)	44.1	2.0 (1.43-2.78)
Obesity	13.8	18.3	1.40 (0.89-2.20)	21.6	1.72 (1.11-2.66)

HTN indicates hypertension; RR, risk ratio.

* Ratio of pre-hypertension group to normotensive group.

# Ratio of hypertension group to normotensive group.

In Table 3 appear the percentages of subjects exhibiting 0, 1, 2, 3, 4 or more additional cardiovascular risk factors according to their blood pressure indices. 7.6% and 7.2% of the normotensive group and 16.3% and 9.3% of the pre-hypertensive group exhibited three and ≥4 additional cardiovascular risk factors, respectively. The corresponding percentages for the hypertensive group were 23.5% and 17.4%, respectively.

**Table 3:** **Clustering**^**1 **^**of Cardiovascular risk factors by blood pressure group.**

**Number of additional Risk factors**	**normal (%)**	**pre-HTN (%)**	**HTN (%)**	**p-value**
0	21.0	8.3	1.9	<0.001
1	38.8	37.7	22.9	<0.001
2	25.4	28.4	34.3	<0.051
3	7.6	16.3	23.5	<0.001
≥4	7.2	9.3	17.4	<0.001

HTN indicates hypertension.

1 Risk factors include diabetes mellitus, hypercholesterolemia (total), high LDL and low HDL cholesterol level of blood, hypertriglyceridemia, smoking, age > 45 years, BMI ≥25 kg/m^2^.

### Multivariate logistic regression

In Table 4 the results of multivariate logistic regression analysis showed that obesity and overweight were the most powerful predictors of pre-hypertension, with an odds ratio (OR) of 2.74 (95% CI, 1.62 to 4.62, p < 0.001) and 2.56 (95% CI, 1.74 to 3.77, p < 0.001), followed by male gender (OR, 1.89; 95% CI, 1.29 to 2.81, p = 0.002). On the contrary, age (p = 0.002) and smoking (p = 0.003) had an inverse effect on pre-hypertension.

**Table 4:** Determinants of prehypertension vs normal blood pressure from multivariable logistic regression model

**Variable**	OR*	**95% CI**	**p-value**
Obesity	2.74	1.62-4.62	<0.001
Overweight	2.56	1.74-3.78	<0.001
Smoking	0.45	0.26-0.76	0.003
Male sex	1.89	1.27-2.81	0.002
Age (year)	0.96	0.95-0.98	<0.001
Diabetes mellitus	1.36	0.66-2.82	0.398
Total	1.07	0.56-2.03	0.833
Hypercholesterolemia			
High LDL level	0.81	0.29-2.23	0.694
Low HDL level	0.92	0.63-1.32	0.65
Hypertriglyceridemia	1.28	0.78-2.12	0.326

*OR = Odds Ratio.

## Discussion

Over the last years increased pre-hypertension rates have been observed and well documented worldwide. However, this area is still not fully investigated. Therefore, the purpose of this study was to further explore this issue focusing on a population-based sample of adults in south Iran.

The results revealed that nearly one third of our population was classified as pre-hypertensive. A higher percentage of the male population - compared to the female population – was found to exhibit pre-hypertension, without, however, significant difference in the percentages. Furthermore, it was also found that obesity and overweight had the strongest association with pre-hypertension but, surprisingly, age and smoking had an inverse effect on pre-hypertension. In addition, it was disclosed that subjects with pre-hypertension had multiple additional cardiovascular risk factors when compared to those with normal blood pressure.

Nontheless, the prevalence of pre-hypertension in the present study (33.7%) was higher when compared with the prevalence estimates reported in Jamaica (31%), in Turkey (14.5%), in Canada (22.0%), in Nigeria (24.8%), in Tailand (32.8%) and in Japan (33.0%). On the contrary, the prehypertension estimate in our sample was lower when compared with the results reported from Taiwan (35.8%), U.S.A. (36.3%) and China (38.4% and 44.1%) [6,7,13,17-23]. However, the present findings contradict the Jamaica Study’s gender specific pattern whereby men (35.0%) rather than women (25.0%) exhibited higher percentages of pre-hypertension [6].

Additionally, it was detected that the prevalence of prehypertension was higher among younger age groups. This finding is in line with the published data from China [7] and Japan [24], but contradicts the results reported in Iran [25] and Nigeria [18] showing an opposite pattern, with a little decrease in persons above 60 years old.

The present study also complements previous reports from Iran demonstrating high prevalence of pre-hypertension. Indeed, results from the Nationwide Survey in Iran between 2004–2005 revealed a high incidence of pre-hypertension in both sexes aged 25–65, i.e., 59.6% in men and 44.5% in women, respectively [25]. Similar results were also reported by [Sahebi et al. 26] where 37.2% of hospital staff in Shiraz, Iran, aged 19 years and over, found to be pre-hypertensive.

However, social and cultural differences, the subject’s characteristics, the age span as well as the methodology used, may account for the observed discrepancies among the previous mentioned studies. Nonetheless, although direct comparisons with other studies are not possible, it is rather self-evident that the prevalence of pre-hypertension is high in both developed and developing countries and in eastern and western populations and, therefore, needs further in depth clarification.

Another main finding revealed that pre-hypertension was associated with an increased prevalence of other cardiovascular risk factors such as age, sex, overweight and obesity. The association of pre-hypertension with multiple risk factors for cardiovascular disease has been described in the Jamaican population [6]. Indeed, the prevalence of hypercholesterolemia, diabetes mellitus, overweight and obesity was found to be greater among persons with pre-hypertension compared to persons who were normotensive in Jamaican population [6]. A number of other studies suggested that persons with pre-hypertension were more likely to have a higher BMI, total cholesterol, triglyceride, higher LDL cholesterol and lower HDL cholesterol level of blood, impaired glucose tolerance, obesity and older age than persons who had normal blood pressure [3,17,18,27]. This clustering of cardiovascular disease risk factors among persons with pre-hypertension suggest the importance of screening for other cardiovascular risk factors in persons who are classified as pre-hypertensive.

Our results confirmed that obesity and overweight are major determinants of pre-hypertension. Similar findings were also found in the Chinese survey, in Japan and in a rural Taiwanese adult population [7,20,24,28-31]. Additionally, in [Sahebi et al. 26] study, the association between pre-hypertension with obesity and overweight was, also, confirmed.

Finally, in our study, the odds of smoking had reverse significant association among pre-hypertensive subjects when compared to normotensive persons. This finding is in line with other studies [6,26,32], but contradict [Kawamoto et al. 24] findings who reported a higher smoking rate in pre-hypertensive subjects when compared with normotensive subjects.

### Limitations

The two BP measurements in one day that was used for our subject classification in the various blood pressure categories, the higher percentage of the female population compared to the male one, lack of data on habitual physical activity and the absence of dietary data could be some limitations that restricted our scientific contribution to the area.

## Conclusions

In conclusion and within the study’s limitations, overweight and obesity were found to be decisively associated with the high prevalence of pre-hypertension in Iranian adults. Additionally, a causative relationship between pre-hypertension and clustering of cardiovascular risk factors was documented. Therefore, primary preventive strategies should focus on reducing overweight and obesity if the future pre-hypertension risk in Iranian population is to be reduced.

## Abbreviations

BMI: Body mass index; CI: Confidence interval; DBP: Diastolic blood pressure; HDL-C: High density lipoprotein cholesterol; IHPS: Iranian health profile survey; JNC7: Seventh report of joint national committee; LDL-C: Low density lipoprotein cholesterol; OR: Odds ratio; SBP: Systolic blood pressure; SD: Standard deviation.

## Competing interest

The authors declare that they have no competing interest.

## Authors’ contributions

RK and SM was involved in data collection, performed data analysis. RK wrote manuscript. SM helped revised manuscript. All authors read and approved the final manuscript.
